# Forestry Applications of Space-Borne LiDAR Sensors: A Worldwide Bibliometric Analysis

**DOI:** 10.3390/s24041106

**Published:** 2024-02-08

**Authors:** Fernando J. Aguilar, Francisco A. Rodríguez, Manuel A. Aguilar, Abderrahim Nemmaoui, Flor Álvarez-Taboada

**Affiliations:** 1 Department of Engineering, CIAIMBITAL Research Center, University of Almería, Carretera de Sacramento s/n, 04120 Almería, Spain; maguilar@ual.es (M.A.A.); an932@ual.es (A.N.); 2 Ministry of Agriculture, Fisheries, Water and Rural Development, Junta de Andalucía, Calle Tabladilla s/n, 41013 Sevilla, Spain; fantonio.rodriguez@juntadeandalucia.es; 3 Department of Mining Technology, Topography and Structures, University of León, 24404 Ponferrada, Spain; flor.alvarez@unileon.es

**Keywords:** space-borne LiDAR, ICESat, GEDI, forest, AGB, remote sensing, bibliometric analysis, Scopus

## Abstract

The 21st century has seen the launch of new space-borne sensors based on LiDAR (light detection and ranging) technology developed in the second half of the 20th century. Nowadays, these sensors offer novel opportunities for mapping terrain and canopy heights and estimating aboveground biomass (AGB) across local to regional scales. This study aims to analyze the scientific impact of these sensors on large-scale forest mapping to retrieve 3D canopy information, monitor forest degradation, estimate AGB, and model key ecosystem variables such as primary productivity and biodiversity. A worldwide bibliometric analysis of this topic was carried out based on up to 412 publications indexed in the Scopus database during the period 2004–2022. The results showed that the number of published documents increased exponentially in the last five years, coinciding with the commissioning of two new LiDAR space missions: Ice, Cloud, and Land Elevation Satellite (ICESat-2) and Global Ecosystem Dynamics Investigation (GEDI). These missions have been providing data since 2018 and 2019, respectively. The journal that demonstrated the highest productivity in this field was “Remote Sensing” and among the leading contributors, the top five countries in terms of publications were the USA, China, the UK, France, and Germany. The upward trajectory in the number of publications categorizes this subject as a highly trending research topic, particularly in the context of improving forest resource management and participating in global climate treaty frameworks that require monitoring and reporting on forest carbon stocks. In this context, the integration of space-borne data, including imagery, SAR, and LiDAR, is anticipated to steer the trajectory of this research in the upcoming years.

## 1. Introduction

Forest ecosystems offer a myriad of ecosystem services and social advantages, encompassing carbon sequestration, wildlife habitat, recreational opportunities, as well as the supply of both wood and non-wood products [1]. Therefore, assessing the value of ecosystem services rendered by forests is crucial for preventing or halting their degradation. This process involves the convergence of scientific, economic, and political domains to implement measures aimed at enhancing their sustainable management. To provide a quantitative figure referring to 1997, Costanza et al. [2] calculated the yearly value of ecosystem services provided by forests to be $4.7 trillion, equivalent to around 15% of the World GNP (gross national product).

Forests encompass approximately 80% of the Earth’s biomass, contributing to 75% of the gross primary productivity in the terrestrial biosphere [3]. They play a pivotal role in the global carbon cycle, constituting a significant component. In fact, forests account for up to 50% of the annual carbon exchange between the atmosphere and the Earth’s land surface [4]. Functioning as natural carbon dioxide sinks, they effectively sequester atmospheric carbon, mitigating fossil fuel emissions at rates reaching about 30% [5].

However, a concerning deficiency exists in the standardization of forest monitoring data, posing a frequent impediment to decision-making processes related to both the economic facets of management activities and the environmental considerations tied to the impacts of climate change and anthropogenic pressures on forest protection by both public and private entities. Traditional terrestrial forest inventory methods that apply sampling procedures at the plot level offer partial results that need to be extended by means of statistical inference to cover the entire forest mass [6,7]. This is an expensive and inefficient procedure that, moreover, is practically unfeasible in remote forest areas with difficult access [8]. In this sense, new forest evaluation and monitoring strategies are demanded, which is closely associated with the development of emerging spatial information technologies headed up to the large-scale evaluation of key variables, such as standing aboveground biomass (AGB). AGB serves as a metric for quantifying the capacity of forests to absorb carbon dioxide, being defined as the sum of living vegetation above the soil, encompassing the stem, stump, branches, bark, seeds, and foliage [9,10].

Remote sensing has been demonstrated to be a cost-effective tool for AGB mapping on large scales, extending to national and even global extents [11]. Given the absence of a remote sensing sensor directly providing AGB information, studies rely on associated variables. Notably, “canopy height” emerges as a crucial variable obtainable from light detection and ranging (LiDAR) sensors, serving as a significant predictor for AGB [12,13,14,15,16,17]. Moreover, it aids in monitoring ecosystem responses to climate variations, forest degradation, land-use changes, and restoration efforts [18,19,20,21].

Historically, LiDAR data for forested areas primarily came from aircraft, limiting its spatial reach. Notably, aerial LiDAR surveys, due to their elevated costs, often prioritize data acquisition in high-value forest areas rather than offering comprehensive coverage at the national or regional levels [22,23]. However, a significant shift has occurred with the emergence of the new generation of space-borne LiDAR sensors, exemplified by missions such as Ice, Cloud, and Land Elevation Satellite-2 (ICE-Sat-2) and Global Ecosystem Dynamics Investigation (GEDI), launched by NASA in 2018. Over the past five years, these sensors have gained prominence for their capability to furnish precise information on vertical vegetation structures on a global scale directly [24].

Satellite LiDAR measurement consists of transmitting laser pulses at a certain frequency from the space laser to Earth. In this way, the laser beam passes through the atmosphere and is scattered by the terrain/canopy surface, generating a weak backscattered echo. The satellite-based LiDAR telescope receives this energy return, and the distance between the sensor and the target on Earth is calculated by converting photoelectric signals and measuring time. This computed distance is processed together with information from the satellite attitude, position, and laser pointing to produce accurate 3D spatial coordinates of the laser footprint point directly [25]. This is the case of GEDI, which marked a pioneering achievement as the inaugural space-borne full waveform LiDAR (photon counting at wavelength λ = 1064 nm), uniquely designed to offer insights into the vertical structure of forest canopies [20,26], with a particular focus on Earth’s temperate and tropical forests. Launched in December 2018, it was installed on the International Space Station (ISS), boasting a footprint of 25 m (with planimetric uncertainty ranging from 8 to 10 m) at intervals of 60 m (along-track) and 600 m (across-track). Some of its derived products are L2A data (LiDAR metrics to determine terrain and vegetation height, vegetation cover, etc.) and L4A (AGB density based on calibrated models). GEDI full waveform data have additionally been employed in generating valuable derived data, including the Leaf Area Index (LAI) product. This product is made available globally through the NASA/USGS Land Processes Distributed Active Archive Center [27]. Due to the confined orbit of the ISS within the latitudes of 51.6° N and S, GEDI is unable to capture a substantial portion of the world’s boreal forests, including regions such as Canada.

Conversely, ICESat-2 faces no constraints in terms of orbit or data acquisition [21], ensuring comprehensive coverage of boreal forest regions. Equipped with the Advanced Topographic Laser Altimeter System (ATLAS), ICESat-2 features a micro-pulse, multi-beam photon-counting LiDAR sensor with a notable pulse repetition frequency of 10 kHz, a footprint of 10.9 ± 1.2 m, and a planimetric uncertainty of around 3.5 ± 2.1 m. These ATL03 data provided by ICESat-2 are employed in generating the land, water, and vegetation elevation product (ATL08 data). This product furnishes canopy height percentiles along 100 m segments by utilizing returned photons categorized as ground, noise, canopy, or top of the canopy [22]. ATL08 supplies terrain elevation metrics such as minimum, maximum, and average terrain heights, along with diverse canopy height metrics encompassing minimum, maximum, average canopy heights, and percentile canopy heights. The precision in retrieving canopy height is contingent on several influencing factors, such as sensor characteristics (strong/weak beams), canopy structure (canopy cover and height), terrain (terrain slope), and external environmental conditions (solar angle and atmosphere scattering) [28]. This space-borne sensor is considered the successor to the first ICESat mission (2003–2009) that used a space-borne LiDAR sensor, the so-called Geoscience Laser Altimeter System (GLAS), to provide large-scale forest biomass and height maps.

Although GEDI and ICESat-2 are the more widely used space-borne LiDAR sensors currently, there are other space-borne laser altimeters worth discussing. This is the case with the Chinese satellites ZY3-02 and Gao Fen-7, launched in May 2016 and November 2019, respectively. ZY3-02 is equipped with high-resolution three-line CCD cameras that provide stereo-mapping capabilities and a multispectral camera. It also counts on a very small and experimental laser altimeter that emits mono-beam discrete pulses with a ground footprint of about 50 m at a sample frequency of only 2 Hz. This low sample frequency implies that the spatial distance along the track between two adjacent footprints is considerable (approximately 3.5 km) [29]. As the ZY3-02 satellite laser altimeter was specifically designed to enhance the elevation accuracy of surveying and mapping satellite image products, it does not transmit back waveform data. Consequently, its applicability as a LiDAR system for forest applications is severely restricted. On the other hand, Gao Fen-7 comes equipped with a stereo mapping camera featuring a two-line array and a laser altimeter system. This configuration allows for the simultaneous capture of stereo images and full-waveform LiDAR data [30]. The dual-beam laser altimeter system on board emits laser pulses at 1064 nm to the ground, operating at an observing frequency of 3 Hz. This results in discrete laser footprints of approximately 20 m in diameter, with along-track and cross-track spacing measuring about 2.4 km and 12.25 km, respectively. The primary limitation of this space-borne LiDAR sensor lies in its low footprint density on the ground. Full waveform data are recorded at 2 GHz with a sampling interval of 7.5 cm [31].

Summing up, and mainly over the past half-decade, space-borne LiDAR has emerged as a pivotal technology, furnishing precise information on the interconnections between biodiversity and ecosystem structure [23]. In this way, space-borne LiDAR data can accurately describe the vertical structure of the forest, although its discrete point distribution of data acquisition hardly supports continuous forest management planning. Overall, it is mandatory to combine space-borne LiDAR data with other imaging remote sensing technologies (Sentinel-1, Sentinel-2, etc.) to obtain wall-to-wall forest height for resource management, policy development, and decision-making at regional or even nationwide studies [27,32]. In this way, the recent launch in August 2022 of the Terrestrial Ecosystem Carbon Monitoring Satellite (TECMS; China State Administration of Forestry and Grassland), coupled with the introduction of a new generation of space-borne active sensors such as Multi-footprint Observation LiDAR and Imager (MOLI; Japan Aerospace Exploration Agency), BIOMASS P-band Synthetic Aperture Radar (SAR) (European Space Agency), and LiDAR Surface Topography (LIST; NASA) (see Table 1), will enhance the array of space-based sensors available for mapping and monitoring extensive forest systems.

## 2. Materials and Methods

### 2.1. Bibliometric Analysis

A traditional bibliometric examination of scholarly documents was executed through co-occurrence analysis applied to metadata within databases based on indicators associated with productivity, quality, and structure [33,34]. 

The indicators associated with the productivity component of the topic “Space-borne LiDAR in forestry applications” were the following: (i) count of indexed articles and conference papers found in the Scopus database on the topic from 2004 to 2022; (ii) exploration of the authors, journals, institutions, and countries associated with the registered documents.

With respect to analyzing the scientific quality component, this study was based on classical scientific impact indicators such as the number of citations, the author/journal h-index [35], and the Scimago Journal Rank (SJR) impact factor of journals [36]. Note that both the h-index and the SJR index take into account both quantity (number of articles) and scientific impact (number of citations). In this way, the SJR index is used to configure the ranking of journals according to the relative scientific impact of the different journals. In doing so, the SJR index considers not only the number of citations received by a journal but also the prestige associated with the source of those citations [36].

Certain structural indicators were formulated to uncover linkages among scientific actors and pinpoint research trends on the topic. This involved utilizing mapping tools such as VOSviewer version1.6.20 (https://www.vosviewer.com/, accessed on 2 February 2024) [37,38,39].

### 2.2. Data Processing

For this study, the Scopus database was selected as it stands as the most extensive repository of peer-reviewed literature [40]. In fact, Scopus includes a greater number of indexed journals compared with Web of Science (WoS) [41]. Notably, approximately 84% of the titles indexed in WoS are present in Scopus, while only 54% of the publications indexed in Scopus are mirrored in WoS [42]. 

The keywords and search string input into Scopus to identify relevant indexed publications for this bibliometric analysis comprised the following:

TITLE-ABS-KEY ((icesat OR “spaceborne lidar” OR gedi) AND (forestry OR forestal OR forests OR forest)) AND (EXCLUDE (PUBYEAR, 1963) OR EXCLUDE (PUBYEAR, 1998) OR EXCLUDE (PUBYEAR, 2003) OR EXCLUDE (PUBYEAR, 2023)) AND (LIMIT-TO (EXACTKEYWORD, “Forestry”) OR LIMIT-TO (EXACTKEYWORD, “Biomass”) OR LIMIT-TO (EXACTKEYWORD, “Vegetation”) OR LIMIT-TO (EXACTKEYWORD, “Aboveground Biomass”) OR LIMIT-TO (EXACTKEYWORD, “Above Ground Biomass”) OR LIMIT-TO (EXACTKEYWORD, “Canopy Heights”) OR LIMIT-TO (EXACTKEYWORD, “Forest Canopy”) OR LIMIT-TO (EXACTKEYWORD, “Ecosystem Dynamics”) OR LIMIT-TO (EXACTKEYWORD, “Canopy Height”) OR LIMIT-TO (EXACTKEYWORD, “Forest Height”) OR LIMIT-TO (EXACTKEYWORD, “Forest”) OR LIMIT-TO (EXACTKEYWORD, “Forest Ecosystem”) OR LIMIT-TO (EXACTKEYWORD, “Forest Structure”) OR LIMIT-TO (EXACTKEYWORD, “Forest Biomass”) OR LIMIT-TO (EXACTKEYWORD, “Forests”) OR LIMIT-TO (EXACTKEYWORD, “Tropical Forest”) OR LIMIT-TO (EXACTKEYWORD, “Forest Canopies”) OR LIMIT-TO (EXACTKEYWORD, “Vegetation Mapping”) OR LIMIT-TO (EXACTKEYWORD, “Vegetation Structure”) OR LIMIT-TO (EXACTKEYWORD, “Tree Height”) OR LIMIT-TO (EXACTKEYWORD, “Boreal Forest”) OR LIMIT-TO (EXACTKEYWORD, “Forest Inventory”) OR LIMIT-TO (EXACTKEYWORD, “Forest Cover”) OR LIMIT-TO (EXACTKEYWORD, “Canopy Height Models”) OR LIMIT-TO (EXACTKEYWORD, “Canopy Architecture”) OR LIMIT-TO (EXACTKEYWORD, “Forest Aboveground Biomass”) OR LIMIT-TO (EXACTKEYWORD, “Forest Canopy Height”) OR LIMIT-TO (EXACTKEYWORD, “Vegetation Height”) OR LIMIT-TO (EXACTKEYWORD, “Vegetation Cover”) OR LIMIT-TO (EXACTKEYWORD, “Forest Management”) OR LIMIT-TO (EXACTKEYWORD, “Canopy Cover”) OR LIMIT-TO (EXACTKEYWORD, “Biomass Estimation”) OR LIMIT-TO (EXACTKEYWORD, “AGB”)) AND (EXCLUDE (LANGUAGE, “Chinese”) OR EXCLUDE (LANGUAGE, “Russian”)).

This search was conducted in 2023, comprising a target study period from 2004 to 2022. An attempt was made to encompass all indexed Scopus items, including document types such as articles, reviews, letters, and conference papers. It is noteworthy that only documents dated up to 2022 were considered to facilitate comparisons across complete 12-month periods [43]. The variables under investigation included the number of publications per year, document type, author, institution, country, subject area, journal, and keywords. 

Several preprocessing tasks were executed to rectify common errors, including duplications, misspellings, and variant names [44]. Following a manual review involving keyword analysis, author scrutiny, and abstract reading, the initial number of documents was reduced to 412, discarding the majority of them.

**Figure 1:**
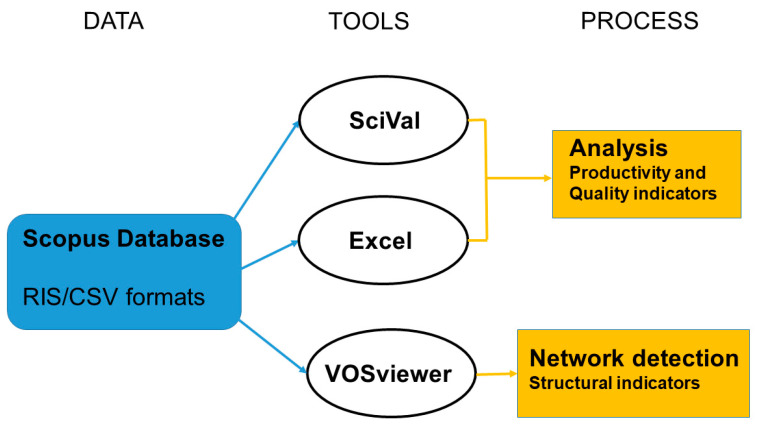
Simplified flowchart of data screening and analysis. RIS is a standardized file format developed by Research Information Systems to enable citation programs to exchange data. CSV is the widely known comma-separated values text file format.

Upon downloading all data in RIS and CSV formats, meticulous manual processing ensued to eliminate duplicates and unrelated elements, resulting in a refined database. This finalized database served as the foundation for generating various tables and figures to streamline the analysis of the collected information. Both Excel (version 2016, Microsoft Corporation, Redmond, Washington, DC, USA) and SciVal (Keep release May 2023, Elsevier) were employed for these tasks. Additionally, VOSviewer played a role in constructing the relevant network maps [39] (see Figure 1). In the case of the co-authorship network, the minimum number of documents from an author was set at five, while the method of analysis used was full counting and association strength with default parameters and merging small clusters. An extra examination of pertinent keywords was conducted using the VOSviewer tool to create a network of co-occurring keywords, shedding light on current research trends within the topic of interest.

## 3. Results

### 3.1. Scientific Production and Characteristics

The search period from 2004 to 2022 yielded 412 documents (access in Scopus comma-separated values format (CSV) at this link (https://drive.google.com/file/d/1tiJAqX1PHDWtjcEUcK7v6OcxzDxUFPoh/view, accessed on 2 February 2024)), including 303 articles (73.5%), 94 conference papers (22.8%), 13 reviews (3.2%), one book chapter (0.2%), and one note (0.2%). Examining the publication trends reveals a growing interest in the research on “Forest applications of Space-borne LiDAR sensor” in recent years (see Table 2). Notably, the majority of documents (51.45%) surfaced within the last four years (2019–2022), with the peak number of publications occurring in 2022 (19.90%). This substantiates that “Forestry applications of space-borne LiDAR sensors” is a burgeoning subject in constant evolution, and it is likely to witness a surge in publications in the upcoming years, particularly with the deployment of the Terrestrial Ecosystem Carbon Monitoring Satellite (China), the Multi-footprint Observation LiDAR and Imager (Japan), and the LiDAR Surface Topography (United States).

**Figure 2:**
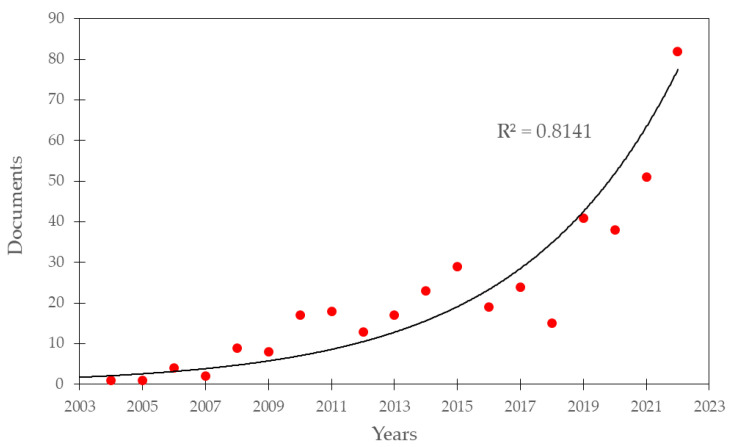
Evolution in the count of published documents (exponential fit with R^2^ = 0.8141).

Figure 2 shows an exponentially increasing relationship (R^2^ = 0.8141) between the number of documents published over the period 2004–2022. This growing interest in this topic among the scientific community can be justified by the urgent need to collect accurate, timely, and large-scale information related to AGB and carbon stocks fixed by forests. The utilization of remote sensing monitoring is deemed essential within the framework of the United Nations Framework Convention on Climate Change, particularly in the context of the strategy for Reducing Emissions from Deforestation and Forest Degradation (REDD) [45]. 

Upon dissecting the bibliometric data showcased in Table 2, a notable escalation is evident. The number of authors (AU) surged from a mere four in 2004 to 588 in 2022, paralleled by the escalation in the number of publications in journals (J) from one in 2004 to 82 in 2022. This heightened scientific impact is further underscored by the extensive references, reaching up to 4383 (number of references; NR), cited in the 82 articles published in 2022, along with the cumulative total citations (TC) amounting to 12,419 across the 412 sample documents collected from 2004 to 2022.

In summary, the forestry applications of space-borne sensors have become an appreciated and valued tool for providing wall-to-wall AGB estimates and information on vertical vegetation structure. This evidence is notably encouraging developed countries to maintain current space-borne LiDAR programs and initiate new ones, not necessarily based on LiDAR technology but also on space-borne SAR sensors (e.g., BIOMASS P-band SAR powered by the European Space Agency). 

### 3.2. Subject Categories and Journals/Conferences

**Figure 3:**
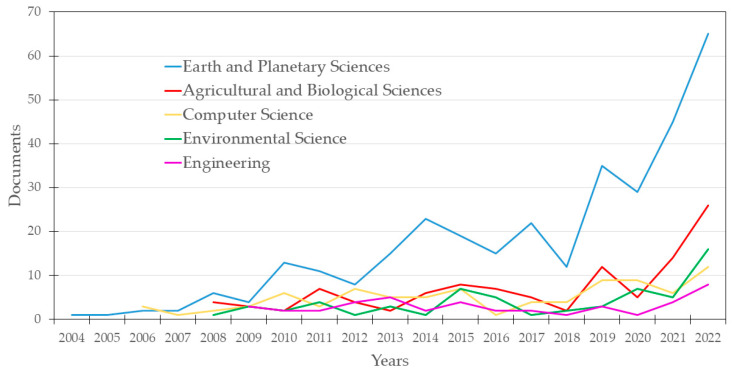
Distribution of publications over time according to Scopus sub-areas category.

All the documents recorded from 2004 to 2022 were categorized into 10 different groups according to the Scopus database. Figure 3 illustrates the temporal evolution of the primary thematic categories assigned to these documents by Scopus. It is worth noting that each document may fall into two or more categories simultaneously. In this way, a substantial portion of the sample was classified across five domains, encompassing up to 85.4% of the published documents. They were Earth and Planetary Sciences (44.6%), Agricultural and Biological Sciences (14.6%), Computer Science (12.0%), Environmental Science (8.4%), and Engineering (5.9%). A consistent pattern emerges toward the end of the period, where the Earth and Planetary Sciences category stands out as predominant, followed by Agricultural and Biological Sciences and Environmental Science.

The 10 journals and conferences indexed in Scopus with the highest number of studies published between 2004 and 2022 are listed in Table 3 and grouped into five periods. The analysis discloses that the most prolific journal in the subject under scrutiny was “Remote Sensing”, securing the top position among leading journals with 90 documents. The inaugural article in this journal (first A in Table 3) surfaced in 2011, although it only claimed the top spot in terms of article count during the fourth sub-period (2015–2018). Following closely in the ranking for publishing articles on “forest applications of space-borne LiDAR sensors” is “Remote Sensing of Environment”, a highly impactful journal that held the premier position in the third sub-period (2011–2014). 

Concerning the impact indicators of journals, “Remote Sensing of Environment” emerges as the journal with the highest total number of citations, boasting 4444 citations. It is significantly ahead of other journals, with “Remote Sensing” accumulating 1454 citations and “International Journal of Remote Sensing” securing 633 citations (Table 3). It is relevant to highlight that “Remote Sensing of Environment” and “International Journal of Remote Sensing” are the journals that published articles with the greatest impact on the topic studied, showing the highest number of citations per article published with 61.7 and 48.7 citations, respectively. The journal “Remote Sensing” presented an average value of 16.2 citations/article, although it is the most important journal if we consider the total number of published documents. 

With respect to quality indices (Table 3), “Remote Sensing of Environment” leads with the highest SJR index of 4.057, trailed by “Environmental Research Letters” with an SJR index of 2.119. The majority of the analyzed documents were positioned within the first quartile based on their SJR index in the 2022 edition, underscoring the elevated quality of the international journals addressing this emerging topic. It is essential to note that an SJR index exceeding 1.5 typically correlates with a highly cited journal.

Looking at the h-index (2022 SJR edition), “Remote Sensing of Environment” presents the highest value (327), followed by “International Journal of Remote Sensing” (195) and “Proceedings of SPIE—The International Society for Optical Engineering” (187). Note that prestigious journals such as “Remote Sensing” (168) and “Environmental Research Letters” (164) are one step below. For “Remote Sensing”, being a relatively recent entrant, it has not accumulated sufficient citations to elevate its h-index at this point.

### 3.3. Countries, Institutions, and Authors

The topic “Forest applications of space-borne LiDAR sensors” was investigated from 2004 to 2022 by researchers from up to 53 countries, demonstrating its great interest worldwide (Table 4). It is important to highlight that a single document could be attributed to multiple countries if the authors hailed from different nationalities, indicating international collaboration. The United States reached the top position with 183 publications, followed by China with 117, and, at a greater distance, the United Kingdom with 44 publications. Note that 47.5% of the publications from the United States were released in the most recent sub-period under examination. Table 4 additionally illustrates the number of documents published per million inhabitants of each country (APC). When considering this productivity ratio relative to the country’s population, the Netherlands claimed the top spot at 0.86, closely followed by Canada at 0.84. 

Concerning the scholarly impact of the scrutinized documents, the United States accumulated the highest number of citations, totaling 8377, followed by China (1788), Canada (1699), and the United Kingdom (1654) in a distinct lower tier. However, when examining the number of citations per publication, Canada secured the top position with 53.09 citations per publication, closely followed by the United States (45.78) and the United Kingdom (37.59).

**Figure 4:**
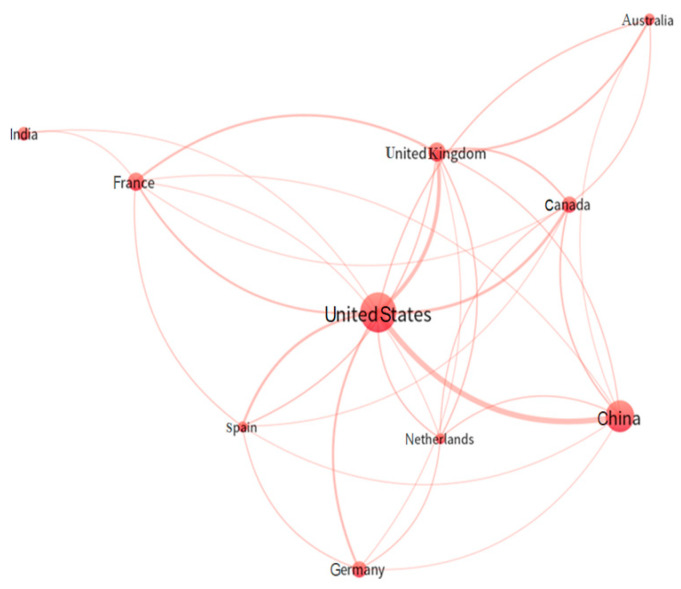
Collaboration network based on co-authorship among countries.

Figure 4 shows a network showcasing international collaboration among publishing countries. The size of each circle corresponds to the number of publications from each country, and the thickness of the lines connecting two collaborating countries is directly proportional to the number of collaborations between them. Note that only one group in red could be extracted, which means that the international network that studies on this topic frequently collaborates, with no clearly independent or isolated research groups existing. This cluster focuses on the US as the country that presents the greatest scientific production on the topic analyzed, also highlighting the very intense collaboration between the US and China and the less important collaboration between the US and the United Kingdom.

Table 5 provides quantitative information on international collaborations among the most prolific countries according to the percentage of publications each country generated in collaboration with others (IC), the count of countries involved in such collaborations (NC), and the principal collaborating countries. Based on these data, it can be affirmed that all countries released documents created through collaborations with other nations. The United States stands out as the country most actively engaged in international collaboration networks, with up to 44.42% of its documents developed in collaboration with other countries. In this way, the United States and France were the countries with the highest number of collaborations with other countries (41 and 39, respectively). It is important to emphasize that there is no clear relationship between international collaboration and scientific impact (measured as total citations by publications segregated by international collaboration (IC) and non-international collaboration (NIC)) (Table 4), varying greatly between countries.

Table 6 outlines the primary production and impact indicators of institutions with the most significant number of publications. It is noteworthy that since some publications involve researchers from different countries and institutions, the total number of publications per country/institution in Table 6 surpasses the count of publications in the overall sample (412). The University of Maryland, College Park, achieved the top position with a total of 77 publications from 2004 to 2022, closely followed by NASA Goddard Space Flight Center (54 documents) and the Chinese Academy of Sciences (51 documents). CIRAD contributed 24 publications but at a greater distance. In terms of scientific impact, measured by the total number of citations, the University of Maryland counted 3701 citations, closely trailed by NASA Goddard Space Flight Center with 3478. However, when examining the average number of citations per publication, Natural Resources Canada emerged as the leader, averaging 80.29 citations per document across 17 articles published in the analyzed period. The California Institute of Technology secured the second position with 79.10 citations per publication and 20 publications. Lastly, the Jet Propulsion Laboratory and the University of Edinburgh exhibited the highest percentage of collaborative studies, with 73.91% and 73.68%, respectively.

**Figure 5:**
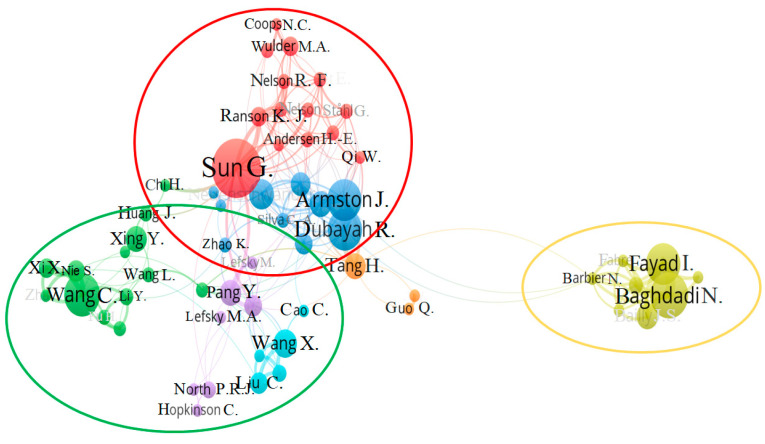
Collaboration network based on co-authorship between authors.

Figure 5 complements Table 6, which illustrates a network map depicting collaborative co-authorship dynamics from 2004 to 2022. Various colors designate groups corresponding to authors who frequently collaborate. The map selectively displays the most interconnected co-authorships. The results shown in Figure 5 have a minimum of six connections. The size of the sphere associated with each author is proportional to the number of published documents. Although several groups were drawn, only three groups have been noted. The group on the right (yellow circle) is made up mainly of French authors (Fayad, I, and Baghdadi, N.), while the group surrounded by a red circle is made up of several subgroups, most of them including authors from the United States (Sung, G., Dubayah, R. and Armston, J.), the United Kingdom (Hankock, S.) and Canada (Coops, N.C., Wulder, M.A.). The last main group is surrounded in green and is mainly composed of authors from China (Wang, C., Pang, Y., and Xing, Y.). Therefore, these three clusters can be considered the three most relevant research networks on the topic analyzed currently in the world. In any case, a highly complex collaboration network is observed within it, with the exception of the French cluster.

Table 7 outlines the 14 most prolific authors within the scope of the topic examined in this study. It encompasses parameters related to both productivity (A) and the scholarly impact of the publications (total citations; TC). Additionally, the table includes pertinent reference data associated with each author, such as author affiliation, country (C), year of initial publication (First P), and the most recent publication (Last P) within this topic.

The initial three authors in the ranking were affiliated with the University of Maryland (College Park) in the United States, each contributing to more than 20 publications. All authors listed in Table 6 hail from the four most prolific countries (Table 3) and are affiliated with nine different institutions. The most productive author turned out to be Sun, G. from the United States. Note that all authors featured in Table 7 remain active in this field, as evidenced by their latest publications spanning from 2019 to 2022.

### 3.4. Keyword Analysis

Table 8 presents the top 20 keywords extensively utilized in the analyzed topic from 2004 to 2022. This period is segmented into five sub-periods to facilitate a comprehensive understanding of keyword usage and its evolution over time. The variables detailed in Table 8 include the keywords’ position in the ranking (R) within each sub-period relative to the total number of keywords in the sample for that period, the frequency of their appearance in publications (A), and the percentage of repetition (%). “Forestry” and “Optical Radar” emerged as the most recurrent keywords throughout the entire study period, consistently claiming top positions in the sub-period rankings. It is important to note that the term “Optical Radar” is another way of referring to the LiDAR sensor. 

In the initial sub-period of 2004–2006, the following two frequently mentioned keywords on the list were “Remote Sensing” and “Biomass”, without including LiDAR in either of its two versions (“Lidar”, more frequent, or “LiDAR”, less frequent). At this early time, it was much more frequent to use the term “Optical Radar” to denote LiDAR. In the second sub-period (2007–2010), researchers’ keyword preferences underwent a slight shift. While “Optical Radar” maintained its position in the second slot in the ranking, there were some alterations in the overall pattern, with keywords such as “Aneroid Altimeters” and “Radio Altimeters” emerging and climbing to third and fourth position, respectively. It is worth clarifying that the aneroid altimeter, also known as an aneroid barometer, is distinct from a LiDAR sensor because it functions as a tool to measure altitude above sea level. Conversely, a radio altimeter determines absolute altitude, indicating the distance above land or water. This determination is based on the principle of reflecting electromagnetic wave pulses off the surface of the Earth or sea. The terms “Lidar” and “LiDAR” also appeared to reach fifth position in the case of the former. The sub-periods spanning 2011–2014 and 2015–2018 marked a transition towards the current configuration of the keyword research framework, also facilitated by the drastic increase in the number of publications on the topic with the design, simulation analysis, and final launching of the space-borne LiDAR sensors ICESat-2 and GEDI. The keyword “Lidar” managed to occupy third/fourth place in the ranking, in clear competition with the keyword “Remote Sensing.” It is necessary to highlight that “Optical Radar” continued to maintain its second position as a frequent physical term to refer to LiDAR technology. The concluding period from 2019 to 2022 continued the trend established in the two preceding sub-periods. Consequently, the final classification aligned closely with the pattern observed in the recent sub-periods, primarily driven by the surge in publications and the consequent expansion in the repertoire of keywords employed in recent years.

**Figure 6:**
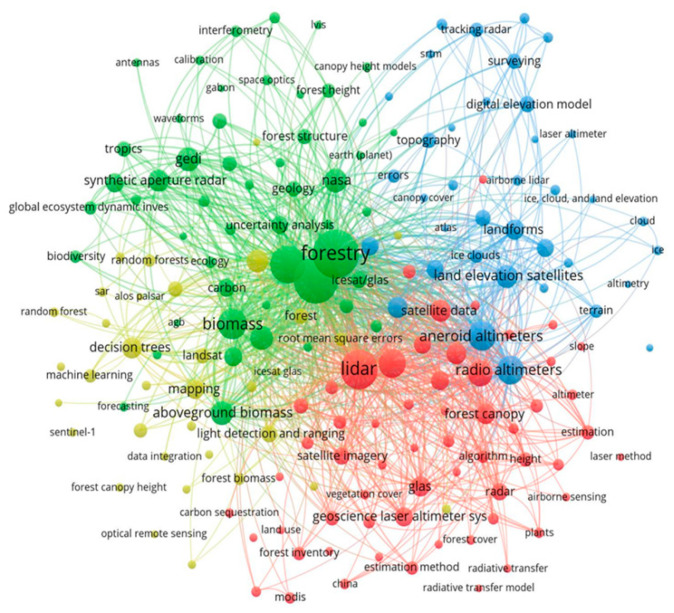
Keywords network on the topic analyzed for the period 2004–2022.

Figure 6 depicts a network map interconnecting various keywords related to the scrutinized publications. Each circle’s size corresponds to the number of publications featuring the respective keyword, while the color designates the cluster to which the keyword belongs, determined by the frequency of co-occurrences. In this sense, the green cluster centers around the term “Forest”, standing out as the cluster with the highest number of connections and exhibiting the closest association with forestry applications. The red cluster presents “Lidar” as its main keyword, which mainly involves data processing methods and complementary technologies. The blue cluster includes keywords such as “Radio altimeters”, “earth elevation satellites”, “digital elevation model”, and “landforms”, being a cluster more focused on geosciences and terrain modeling. The yellow cluster grouped keywords mainly related to machine learning methods and other data sources (Landsat, Sentinel-1, ALOS PALSAR).

## 4. Discussion

First of all, it is necessary to clarify the objective of this study, which focuses on carrying out a bibliometric analysis that provides a quantitative framework to understand the evolution and current situation of the topic “Forest applications of space-borne LiDAR sensors” from 2004 to 2022. With this objective in mind, we analyzed several objective indicators grouped into five categories: (i) evolution of scientific production, (ii) main actors involved, (iii) information dissemination (indexed journals/conferences and keywords), (iv) scientific impact, and (v) scientific collaboration networks. It is noteworthy that bibliometric methods have become an integral component of research evaluation methodology, particularly in the scientific and applied domains [46]. Therefore, it would be a mistake to confuse the approach applied in this study with the typical approach used when writing a review article. In this study, we conducted a bibliometric analysis utilizing relevant statistical data, providing a suitable approach to assess the scientific production within the studied topic. The outcomes are presented for the benefit of researchers, policymakers, and other stakeholders [46].

The topic under study has proven to be an emerging research discipline in the field of remote sensing only in the last five years due to two concomitant facts. First, the most important space LiDAR sensors, i.e., ICESat-2 and GEDI, were launched in 2018 and became fully operational in 2019. Before that, only ICESat-1 (launched in January 2003 and operated until February 2010) and some experimental laser altimeters were available (e.g., ZY3-02 and Gao Fen-7). Without a doubt, the launch into orbit in 2018 of the two LiDAR sensors mentioned has marked a great change in the evolution of AGB estimation using LiDAR technology. Second, the predominant research themes in space-borne LiDAR studies have consistently revolved around forest inventory and forest productivity [24] because there is a clear need to know what carbon stock is fixed by forests. In other words, it is evident that the pivotal role of effectively monitoring forests on a large scale is crucial for adapting to climate change [1,47]. Note that the terrestrial carbon budget due to land use change and carbon absorption by forests is more uncertain than others, and this uncertainty is mainly caused by the difficulty in measuring forests globally [48]. 

These are the two reasons, especially the first of those listed, why scientific production on the topic analyzed has only increased significantly during the last five years. This could elucidate the relatively modest count of indexed Scopus documents found during the 2004–2022 period (412 documents, also considering conference papers). Concurrently, it is vital to acknowledge that citations not only require time to accrue but also persist in accumulating over time [49]. Indeed, certain studies have asserted that documents necessitate a minimum of two to three years post-publication to amass sufficient citations, ensuring the reliability of bibliometric indicators [50,51]. Under this perspective, the pioneering nations in this domain, namely the United States and China, with 8377 and 1788 accumulated citations, respectively, along with a select few institutions such as the University of Maryland and NASA Goddard Space Flight Center in the United States, the Chinese Academy of Sciences in China, and CIRAD in France, are poised to constitute the core of this topic for the foreseeable future.

What can we expect in the coming years? There has been a noticeable and escalating recognition in the 21st century regarding the environmental consequences linked to deforestation and biodiversity degradation. Moreover, there is an urgent need to comprehend the impacts of climate change attributed to the expanding global population and evolving consumption patterns. [52]. In this context, carbon sequestration, a pivotal element in the climate equation, will assume a significant role in mitigating pollution and gauging an area’s capacity to generate biomass. Given the costliness and inefficiency of traditional approaches for creating inventories of production units through in-situ studies (ground monitoring), there is a shift toward semi-automatic methods utilizing machine learning and deep learning approaches. These methods leverage geo-referenced digital information often extracted from the expanding realm of satellite imagery [53,54]. In this way, the new generation of space-borne LiDAR sensors, in addition to ICESat-2 and GEDI, will notably contribute to expanding the current capabilities to map and monitor forest systems at very large scales.

Within this new generation of space-borne LiDAR sensors, three relevant programs stand out due to their great potential. This is the case of the Terrestrial Ecosystem Carbon Monitoring Satellite, launched in August 2002, which is the first Chinese satellite focused mainly on the forestry sector. It is equipped with a multi-beam pulse LiDAR (25–30 m footprint and 250 m along-track spacing) and high-resolution multi-angle multispectral cameras to estimate canopy height and AGB [55]. The second sensor to take into account in the coming years would be the Multi-footprint Observation LiDAR and Imager (MOLI; Japan Aerospace Exploration Agency). It will mark the inaugural forest observation system to concurrently employ LiDAR and an imager, enabling multiple footprint observations simultaneously. This sensor will be installed aboard the International Space Station and will consist of a 1064 nm dual-beam LiDAR with a pulse repetition frequency of 150 Hz for yielding two parallel paths separated by 50 m on the ground and footprints of 25 m at 50 m intervals along track [56]. Note that it is possible to measure the slope angle by analyzing triangular triplets of footprints to correct canopy height and ground elevation errors due to slope. Previous simulations in the MOLI system have expected to obtain estimates of canopy height with an error of ±3 m (canopy height less than 15 m) and a relative error of ±20% (canopy height greater than 15 m). In the same way, AGB will be estimated with an error of ± 25 t/ha (AGB below 100 t/ha) and ±25% relative error (AGB above 100 t/ha) [56]. Finally, the LiDAR Surface Topography (LIST; NASA), which is scheduled to launch in 2025, will mount a very powerful photon-counting detection system with 1000 beams to achieve footprints of 5 m in diameter separated by about 0.7 m on the ground (swath mapping). This system will allow obtaining accurate and global topographic information of 5 m grid size and surface elevation changes in forests, lakes, and ice caps [25].

In recent years, there has been a discernible shift towards research endeavors focused on integrating data from diverse satellite remote sensors (optical imagery, SAR, LiDAR, and hyperspectral). Indeed, there is a clear synergy between different sources of remote sensing data. Optical data offer valuable insights into vegetation status, while SAR excels in all weather conditions, providing details on the physical structure of vegetation. Meanwhile, LiDAR furnishes accurate elevation data without encountering the saturation issues that optical images and SAR data show in heavily vegetated areas and tall canopies. This kind of multi-sensor synergistic approach can effectively predict the forest canopy height and AGB [57]. This line of spatial data fusion research, coupled with the increasing use of deep learning algorithms in which optical and SAR data play the role of predictors and height measurements from spatial LiDAR sensors serve as calibration data (e.g., [58]), will likely boost large-scale forest monitoring in the coming years.

## 5. Conclusions

The results obtained in this bibliometric analysis showed that the scientific communications selected from the Scopus database on the topic analyzed were mostly published in the format of articles (73.5%) and conference papers (22.8%), which is the usual way of disseminating scientific knowledge in well-established research disciplines. The number of documents per year has increased exponentially, especially in the last five years in which both ICESat-2 and GEDI sensors have been gathering data. Indeed, a substantial portion of the publications (51.45%) emerged within the most recent four years (2019–2022), with the peak volume occurring in 2022 (19.90%). This indicates that the entry into service of the new generation of space-borne LiDAR sensors, which incorporate improved technology for capturing high-resolution LiDAR data, will facilitate the emergence of new methods and approaches for large-scale forest mapping.

Regarding the scientific impact of the journals involved in the dissemination of knowledge about this field during the period 2004–2022, “Remote Sensing of Environment” turned out to be the journal with the highest total number of citations (4444 citations), followed at a great distance by “Remote sensing” (1454) and “International Journal of Remote Sensing” (633). Similarly, “Remote Sensing of Environment” and “International Journal of Remote Sensing” emerged as the journals contributing significantly to the discussed subject, displaying the highest citation rates per published article (61.7 and 48.7 citations/article, respectively). Notably, the journal “Remote Sensing” led in terms of document output, publishing up to 90 articles.

With respect to the most productive countries, The United States took the lead in document production, with the University of Maryland, College Park, emerging as the foremost research institution in this area, highlighting notable collaborations with various Chinese institutions. In this way, the primary global hubs for collaborative research were the United States, China, and France, forming the three major clusters in the production of research. It should be noted that high-resolution space-borne LiDAR Earth observation has proven to be a cutting-edge engineering research direction internationally, and the United States, primarily through NASA programs, has in recent years led the way in the research content and direction of development of future Earth observation. In any case, China and Japan are also developing sensors (i.e., TECM and MOLI), which will contribute to them probably sharing the lead with the United States in the coming years.

In summary, the increasing number of publications from 2004 to 2022 indicates that the subject under study can be classified as a trending research topic, garnering growing interest on a global scale due to the urgent need to collect accurate, timely, and large-scale information related to AGB and carbon stocks fixed by forests. The extensive monitoring is deemed crucial within the United Nations Framework Convention on Climate Change strategy, specifically in the context of Reducing Emissions from Deforestation and Forest Degradation. The escalating need for data on the distribution and temporal dynamics of carbon sequestration in forests, crucial for estimating climate change impacts, is likely to propel further research in this direction in the upcoming years.

## Figures and Tables

**Table 1:** Some specifications of major space-borne LiDAR sensors.

Satellite/Sensor	Launch Year	Country	Number of Beams	Footprint (m)	Sampling Interval (m) ^1^	Type of Detection	Main Applications
ICESat/GLAS	2003	US	1	70	170	Pulse	Ice sheet mass balance and global sea level, distribution of clouds and aerosols, land topography, and vegetation cover
ZY3-02	2016	China	1	50	3500	Pulse	Experimental laser altimeters are very limited for forestry applications
ICESat-2/ ATLAS	2018	US	6	∼11 (<17 m)	0.7	Photon counting	Ice sheets mass balance, atmosphere, land topography, and vegetation cover
GEDI ^2^	2018	US	8	25	60	Pulse	Forest biomass monitoring, water resource management, and geomorphometry
Gao Fen-7	2019	China	2	∼20	2900	Pulse	Capturing elevation control points
Terrestrial Ecosystem Carbon Monitoring Satellite	2022	China	5	∼25	200	Pulse	Forest biomass and carbon monitoring, water level measurement
Multi-footprint Observation Lidar and Imager (MOLI) ^2^	To be launched	Japan	2	25	50	Pulse	Canopy height and forest biomass
LiDAR Surface Topography (LIST)	To be launched	US	1000	5	0.7	Photon counting	High-resolution global topography and vegetation structure

^1^ Along-track direction. ^2^ Aboard the International Space Station.

**Table 2:** Key attributes of documents pertaining to the topic between 2004 and 2022.

Years	A	NR	NR/A	TC	TC/CA	AU	AU/A	J	C
2004	1	0	0.0	13	13.0	4	4.0	1	2
2005	1	14	14.0	527	527.0	8	8.0	1	3
2006	4	35	8.8	31	7.8	16	4.0	2	4
2007	2	30	15.0	246	123.0	11	5.5	2	4
2008	9	246	27.3	986	109.6	39	4.3	7	7
2009	8	262	32.8	316	39.5	35	4.4	7	11
2010	17	411	24.2	932	54.8	56	3.3	14	7
2011	18	708	39.3	1543	85.7	77	4.3	16	7
2012	13	436	33.5	862	66.3	94	7.2	10	11
2013	17	564	33.2	527	31.0	101	5.9	15	14
2014	23	898	39.0	750	32.6	131	5.7	23	15
2015	29	1425	49.1	726	25.0	160	5.5	24	17
2016	19	961	50.6	587	30.9	101	5.3	17	12
2017	24	1263	52.6	1002	41.8	124	5.2	24	15
2018	15	791	52.7	308	20.5	105	7.0	14	13
2019	41	1948	47.5	1238	30.2	244	6.0	41	19
2020	38	1988	52.3	758	19.9	237	6.2	36	19
2021	51	2892	56.7	810	15.9	290	5.7	48	20
2022	82	4383	53.5	257	3.1	588	7.2	82	38
Total	412	19,255	46.7 *	12,419	30.1 *	2421 **	5.9 *	384	54 **

A: Annual number of total documents; NR: Number of references in total publications; NR/A: Annual number of references per publication; TC: Annual number of citations in cumulative publications; TC/CA: Annual total citations per cumulative publication, AU: Annual number of authors; J: Annual number of publications in journals; C: Annual number of countries. *: Average value. **: Total participants.

**Table 3:** Characteristics of the top 10 most productive Scopus-indexed journals and conferences from 2004 to 2022.

Journal/Conference	A	SJR	C	TC	TC/A	h	1st A	A (R)				
								2004–2006	2007–2010	2011–2014	2015–2018	2019–2022
Remote Sensing	90	1.136 (Q1)	Switzerland	1454	16.2	168	2011	0	0	6 (3)	16 (1)	68 (1)
Remote Sensing of Environment	72	4.057 (Q1)	United States	4444	61.7	327	2008	0	5 (2)	15 (1)	11 (2)	41 (2)
International Geoscience and Remote Sensing Symposium (IGARSS)	42	0.255	United States	109	2.6	79	2004	3 (1)	3 (3)	11 (2)	4 (7))	21 (3)
IEEE Journal of Selected Topics in Applied Earth Observations and Remote Sensing	16	1.264 (Q1)	United States	339	21.2	114	2013	0	0	5 (4)	7 (3)	4 (7)
International Journal of Remote Sensing	13	0.732 (Q1)	United Kingdom	633	48.7	195	2008	0	6 (1)	2 (8)	5 (4)	0
International Archives of the Photogrammetry, Remote Sensing and Spatial Information Sciences—ISPRS Archives	12	0.274	Germany	76	6.3	82	2006	1 (2)	2 (5)	1 (10)	4 (6)	4 (8)
International Journal of Applied Earth Observation and Geoinformation	12	1.628 (Q1)	Netherlands	297	24.8	120	2010	0	1 (6)	2 (7)	4 (5)	5 (6)
IEEE Geoscience and Remote Sensing Letters	11	1.284 (Q1)	United States	129	11.7	138	2013	0	0	3 (6)	0	8 (4)
Proceedings of SPIE—The International Society for Optical Engineering	10	0.166	United States	12	1.2	187	2009	0	3 (4)	5 (5)	2 (8)	0
Environmental Research Letters	8	2.119 (Q1)	United Kingdom	111	13.9	164	2011	0	0	1 (9)	0	7 (5)

A: number of total publications; SJR: Scopus Journal Ranking (2022); C: country; TC: number of citations in total publications; TC/A: total citations per publication; h: journal h-index (year 2022); 1st A: first publication research by journal; R: ranking position (1st Documents, 2nd SJR).

**Table 4:** The 10 most productive countries according to research on “Forest applications of space-borne LiDAR” (2004–2022).

Country	A	APC	TC	TC/A	Documents (A)				
					2004–2006	2007–2010	2011–2014	2015–2018	2019–2022
United States	183	0.54	8377	45.78	4	22	33	37	87
China	117	0.08	1788	15.28	0	11	16	28	62
United Kingdom	44	0.66	1654	37.59	0	4	9	12	19
France	39	0.58	595	15.26	0	0	13	10	16
Germany	33	0.40	370	11.21	0	1	6	4	22
Canada	32	0.84	1699	53.09	0	5	6	7	14
India	21	0.01	180	8.57	0	0	2	4	15
Australia	16	0.64	307	19.19	1	1	1	4	9
Netherlands	15	0.86	381	25.40	1	3	2	1	8
Spain	15	0.32	185	12.33	0	0	1	2	12

A: number of total publications; APC: number of documents per 1 million inhabitants; TC: number of citations in total publications; TC/A: total citations per publication.

**Table 5:** International research collaboration on the topic between countries from 2004 to 2022.

Country	IC%	NC	Main Collaborators (A)	TC/A	
				IC	NIC
United States	44.42%	41	China (42), United Kingdom (22), Canada (11), Spain (10), Germany (9), Gabon (8), Norway (8)	48.01	16.35
China	28.40%	31	United States (42), Canada (5), Netherlands (3), United Kingdom (3)	16.30	35.95
United Kingdom	10.68%	34	United States (22), France (8), Australia (7), Gabon (7)	39.82	29.29
France	9.47%	39	United Kingdom (8), Brazil (7), United States (7), Gabon (6)	16.49	31.87
Germany	8.01%	33	United States (9), Netherlands (3), Norway (3), Spain (3)	12.67	31.96
Canada	7.77%	32	United States (11), United Kingdom (6), China (5), Norway (4)	55.47	28.30
India	5.10%	21	Estonia (1), France (1), Italy (1), United States (1)	9.29	31.55
Australia	3.88%	16	United Kingdom (7), United States (4), Canada (3), China (2)	21.00	30.79
Netherlands	3.64%	15	United Kingdom (4), United States (4), Austria (3), Canada (3)	27.53	30.52
Spain	3.64%	15	United States (10), Portugal (4), United Kingdom (4), France (3)	15.73	30.97

IC: international collaborations; NC: total number of international collaborators; TC/A: total citations per publication; NIC: non-international collaborations.

**Table 6:** Characteristics of the main institutions on the topic analyzed from 2004 to 2022.

Nº	Institution	C	A	TC	TC/A	IC (%)
1	University of Maryland, College Park	USA	77	3701	48.06	68.83%
2	NASA Goddard Space Flight Center	USA	54	3478	64.41	59.26%
3	Chinese Academy of Sciences	China	51	1241	24.33	54.90%
4	CIRAD	France	24	393	16.38	62.50%
5	Jet Propulsion Laboratory	USA	23	1808	78.61	73.91%
6	INRAE	France	23	328	14.26	52.17%
7	State Key Laboratory of Remote Sensing Science	China	23	561	24.39	65.22%
8	AgroParisTech	France	22	299	13.59	50.00%
9	Territoires, Environnement, Télédétection et Information Spatiale	France	22	299	13.59	45.45%
10	University of Chinese Academy of Sciences	China	21	450	21.43	57.14%
11	California Institute of Technology	USA	20	1582	79.10	70.00%
12	The University of Edinburgh	United Kingdom	19	704	37.05	73.68%
13	CNRS Centre National de la Recherche Scientifique	France	18	290	16.11	55.56%
14	Natural Resources Canada	Canada	17	1365	80.29	52.94%
15	IRD Centre de Montpellier	France	17	248	14.59	52.94%

C: country; A: number of total publications per institution; TC: number of citations in total publications; TC/A: total citations per publication; IC (%): percentage of international collaborations (number of international collaborations/A).

**Table 7:** The most productive authors on the topic analyzed during the period 2004–2022.

Authors	A	TC	h-Index	C	Affiliation	First P	Last P
Sun, G.	27	1151	39	United States	University of Maryland, College Park	2004	2019
Dubayah, R.	22	957	56	United States	University of Maryland, College Park	2006	2022
Armston, J.	21	857	24	United States	University of Maryland, College Park	2015	2022
Baghdadi, N.	21	274	53	France	Irstea, Antony	2013	2022
Fayad, I.	19	266	13	France	Université de Montpellier	2014	2022
Hancock, S.	16	492	25	United Kingdom	The University of Edinburgh	2012	2022
Tang, H.	15	698	18	Singapore	National University of Singapore	2014	2022
Duncanson, L.	14	473	23	United States	University of Maryland, College Park	2017	2022
Neuensch-wander, A.	14	784	23	United States	The University of Texas at Austin	2010	2022
Wang, C.	13	216	30	China	Aerospace Information Research Institute, Beijing	2011	2022
Bailly, J.S.	12	234	26	France	AgroParisTech	2014	2022
Pang, Y.	12	424	24	China	Chinese Academy of Forestry	2007	2019
Xing, Y.	12	134	11	China	Northeast Forestry University	2008	2022
Nie, S.	10	177	19	China	Aerospace Information Research Institute	2015	2022

A: Documents; TC: Total Citations; C: Country; First P: first-year publication; Last P: last year publication.

**Table 8:** The top 20 most frequently used keywords on the topic analyzed from 2004 to 2022.

	2004–2022		2004–2006			2007–2010			2011–2014			2015–2018			2019–2022		
Keywords	A	%	R	A	%	R	A	%	R	A	%	R	A	%	R	A	%
Forestry	282	68.4%	1	6	100%	1	23	64%	1	40	56%	1	59	68%	1	154	73%
Optical Radar	252	61.2%	2	4	67%	2	23	64%	2	40	56%	2	43	49%	2	142	67%
Remote Sensing	173	42.0%	4	3	50%	6	16	44%	3	36	51%	4	31	36%	3	87	41%
Lidar	155	37.6%		0	0%	5	17	47%	4	32	45%	3	31	36%	4	75	35%
Biomass	119	28.9%	5	2	33%	8	14	39%	6	22	31%	6	29	33%	6	52	25%
Aneroid Altimeters	106	25.7%	11	1	17%	3	21	58%	9	18	25%	5	30	34%	16	36	17%
Vegetation	102	24.8%	7	2	33%	7	15	42%	10	18	25%	9	25	29%	12	42	20%
Radio Altimeters	99	24.0%	64	1	17%	4	21	58%	12	16	23%	8	28	32%	20	33	16%
ICESat	91	22.1%	48	1	17%	24	6	17%	7	19	27%	7	28	32%	15	37	17%
Aboveground Biomass	79	19.2%	9	1	17%	16	7	19%	16	13	18%	11	17	20%	13	41	19%
Ecosystems	72	17.5%	6	2	33%	61	3	8%	46	6	8%	14	17	20%	7	44	21%
GEDI	67	16.3%		0	0%		0	0%		0	0%	99	3	3%	5	64	30%
NASA	66	16.0%	59	1	17%	74	3	8%	32	9	13%	38	9	10%	8	44	21%
Satellite Data	65	15.8%	68	1	17%	34	5	14%	15	14	20%	21	13	15%	21	32	15%
Mean Square Error	60	14.6%		0	0%	122	2	6%		2	3%	20	13	15%	10	43	20%
Land Elevation Satellites	58	14.1%		0	0%	32	5	14%	43	7	10%	27	11	13%	19	35	17%
Satellites	56	13.6%	69	1	17%	9	14	39%	8	19	27%	34	10	11%	62	12	6%
Synthetic Aperture Radar	56	13.6%		0	0%		0	0%	70	4	6%	35	10	11%	11	42	20%
GLAS	55	13.3%		0	0%	22	6	17%	24	11	15%	10	22	25%	50	16	8%
LiDAR	55	13.3%		0	0%	17	7	19%	17	13	18%	17	14	16%	36	21	10%

A: Documents; R: Ranking of the total Keywords in this period; %: In how many documents this keyword appears among all documents in this period.

## Data Availability

The searched period on the analyzed topic during the period 2004–2022 yielded 412 documents (Scopus database). These documents can be accessed in Scopus comma-separated values format (CSV) at this link: https://drive.google.com/file/d/1tiJAqX1PHDWtjcEUcK7v6OcxzDxUFPoh/view (accessed on 2 February 2024).
